# Elemental Analysis and Chemometric Assessment of Edible Part and Peel of Mango Fruits (*Mangifera indica* L.) †

**DOI:** 10.3390/foods14173096

**Published:** 2025-09-03

**Authors:** Michaela Zeiner, Ema Mihalić, Iva Juranović Cindrić, Ivan Nemet, Heidelore Fiedler

**Affiliations:** 1 Man-Technology-Environment Research Centre, School of Science and Technology, Örebro University, Fakultetsgatan 1, 70182 Örebro, Sweden; 2 Department of Chemistry, Faculty of Science, University of Zagreb, Horvatovac 102a, 10000 Zagreb, Croatia; 3 Organic and Biological Analytical Chemistry Group, MolSys Research Unit, University of Liège, 4000 Liège, Belgium

**Keywords:** ICP-MS, metals and metalloids, correlation analysis, classification, mango, microwave-assisted digestion

## Abstract

Mango (*Mangifera indica* L.) is a very popular tropical drupe that can be consumed fresh or dried. It is rich in essential nutrients such as vitamins, dietary fibre, and minerals, as well as biologically active substances, with a positive effect on health. However, it can also contain potentially toxic elements, which justifies the need of properly investigating this food product. Commercially available samples of dried mango, as well as the mesocarp and peel of fresh mango, were analysed. Prior to the multi-element analysis by inductively coupled plasma mass spectrometry (ICP-MS), the microwave-assisted sample digestion method using various reagents and reagent mixtures was optimised, showing that a mixture of nitric acid and hydrogen peroxide gave the best recoveries. The results obtained were processed by chemometric methods. The content of elements in the peel was higher than in the mesocarp. The macroelements Ca, K, Mg, and Na were found in the largest proportion, and the micronutrients present in significant quantities were Cu, Zn, and Mn (>3 mg/kg), while toxic elements, which according to the guidelines of The European Food Safety Authority) would represent a danger to human health, were not found in mass fractions above the permissible values.

## 1. Introduction

Fruits are rich in essential nutrients such as vitamins, essential elements, and dietary fibre, along with biologically active compounds that, while not nutritionally valuable, positively impact health. Especially as dried fruits, such as apples, apricots, dates, figs, peaches, pears, prunes, raisins, and cranberries, they provide these essential nutrients, phytochemicals, and antioxidants in smaller serving sizes, with potential health benefits since the dietary needs are faster met. Dried fruits serve as a good source of natural sugars, particularly fructose, which provides energy to the body. Dried fruit contains approximately three times the amount of dietary fibre compared to fresh fruit. The recommended daily intake of dietary fibre ranges from 25 g to 38 g, depending on gender and age. Dried fruit is high in dietary fibre (3.7 g/100 g–9.8 g/100 g), meaning that a single portion (40 g) represents about 9% of the total recommended daily intake, depending on the fruit [1,2]. Fibre can aid digestion, promote a feeling of fullness, help regulate blood sugar levels, and positively affect heart health. The composition of dried fruit varies depending on the drying method as well as the origin and variety of the fruit. As consequence of drying, also the content of elements is higher in dried fruit compared to fresh. While their levels greatly depend on the type of fruit, the most common elements found include potassium, magnesium, calcium, zinc, iron, copper, manganese, and selenium. These elements are crucial for the normal functioning of the human body, as they cannot be synthesised and thus must be taken up through food or medicinal preparations. Each element has a specific role in the body and an impact on health, with most essential elements having recommended dietary allowances (RDA), which can be met, for example, by consuming small amounts of dried fruit [1,3]. However, it is important to monitor the levels of certain elements, such as potassium, which is abundant in prunes and apricots, as excessive intake can adversely affect individuals with kidney problems [4].

Another important group of nutrients in fruit are vitamins, including water-soluble vitamins (betaine, choline, folic acid, niacin, pantothenic acid, pyridoxine, riboflavin, thiamine, and vitamin C), as well as fat-soluble ones (A, E, and K). Vitamins are crucial for cellular energy production, growth, and division. They play numerous roles in the body, including preserving vision, strengthening the immune system and bones, collagen production, and healing damaged cells. The average daily diet often lacks sufficient vitamins, making dried fruit an excellent way to supplement them. The specific vitamins found in dried fruit depend on the type of fruit. For instance, prunes are very rich in vitamin K, while dried mango is high in vitamin A. The drying process can lead to the loss of heat-sensitive vitamins such as vitamin C, making fresh fruit a better choice if these vitamins are a priority [1,5].

In addition to essential nutrients, dried fruit is rich in nutrients that, while not considered essential, are studied for their potential health benefits. These include phytochemicals, which are natural compounds found in plant-based foods. Phytochemicals may have antioxidant, anti-inflammatory, and other bioactive properties. The most common phytochemicals found in dried fruit include phenolic acids, flavonoids, phytoestrogens, and carotenoids. Dried prunes are an excellent source of phenolic compounds with antioxidant activity, reducing the harmful effects of free radicals in the body and potentially protecting cells from oxidative damage associated with various chronic diseases and aging. On the other hand, dried mango contains carotenoids like beta-carotene, which is important for maintaining healthy skin and vision [1,2,6,7].

Mango (*Mangifera indica* L.) is a tropical fruit belonging to the cashew family, *Anacardiaceae*. It is also classified as a drupe because its juicy, soft mesocarp surrounds a stone that contains the seed. The mango tree is a long-living evergreen plant that can grow up to 30 m tall. Native to Southeast Asia, it has been cultivated for thousands of years on the Indian subcontinent due to its excellent fruits, which are a rich source of fibre, vitamins A and C, essential amino acids, and numerous phytochemicals. It is often called the “king of fruits” because of its numerous health benefits [8,9].

Mango has been cultivated for over 4000 years, with origins in South Asia—especially present-day India, Bangladesh, and Myanmar. Ancient Hindu texts mention its use, reflecting its long-standing value for taste and nutrition. Trade and travel spread mango worldwide: Buddhist monks gifted it, Persian traders carried it to the Middle East and Africa, and the Portuguese introduced it to Europe in the 15th century. Spanish traders later brought it to the Americas, establishing it in tropical and subtropical regions across the globe [10].

Today, mango is one of the most widely consumed fruits, and it is cultivated in many countries with suitable climates, including India, China, Thailand, Indonesia, the Philippines, Mexico, and various countries in Africa and the Caribbean. Stable isotope ratios and elemental composition profiles, with Cd, Se, Cs, As, Pb, and Ba being the main indicators, can accurately distinguish Spanish mangoes from other varieties, contributing to the establishment of a Spanish protected geographical indication [11].

Mango is a beloved fruit worldwide, known for its sweet, juicy flesh and its versatile use in cooking [12]. Already in the 1990s, its role as a source of trace elements was reported [13]. Also, derived products, such as mango jam, show trace metal content in a nutritional relevant range of 0.1 mg/kg to 0.5 mg/kg for Fe, Cu, Zn, Pb, Sn, Cr, and Cd [14].

Mango flesh, peel, and kernel contain high nutritional value and bioactive compounds, with potential nutraceutical significance for enhancing human nutrition and health. The flesh is rich in Ca, Na, Cu, Fe, P, Mn, Mg, Zn, B, and Se, with their mass fractions ranging from 0.6 mg/kg to 10.6 mg/kg [15].

Mango fruit contains diverse phytochemicals, including gallic acid substituted compounds, quercetin, hydrolysable tannins, and fatty acids, potentially contributing to human health and nutrition [16].

Fresh mango spoils quickly after harvest, but drying extends its shelf life by removing water (about 85% of its mass), which slows microbial growth while preserving nutrients. Drying also reduces its mass significantly—100 kg of fresh mango yields about 17 kg dried—making storage and transport much easier [17].

Drying is one of the oldest food preservation methods, first practiced through sun drying, which is still used today despite its limitations. Over time, new technologies such as hot air ovens, microwaves, vacuum, infrared, osmotic dehydration, and freeze-drying have emerged, with recent innovations including ultrasound, pulsed electric fields, and high-pressure techniques. These methods, often used alone or in combination, are chosen based on product type; quality; cost; and desired characteristics like colour, aroma, and nutrition [18,19,20,21].

Apart from the edible mango flesh, also its peel and kernels are important nutrient sources. Mango peels contain valuable components such as polyphenols, dietary fibre, carotenoid, vitamins C and E, and enzyme activities, making them a valuable waste product [22].

Mango kernels are a rich source of nutrients (e.g., Ca, Mg, K, P) and bioactive compounds, making them a potential food ingredient and eco-friendly solution for waste management and environmental pollution [23].

This study investigated the multi-element composition of commercially available dried mango and fresh mango samples, including both mesocarp (flesh) and peel, to assess their nutritional and potentially toxic element content. Multi-element analysis was carried out using inductively coupled plasma mass spectrometry (ICP-MS) to quantify macroelements (elements present at >100 mg/kg, such as calcium, potassium, magnesium, and sodium) and micronutrients (such as copper, zinc, and manganese). This study also aimed to detect any potentially toxic elements that could pose a health risk according to the guidelines set by the European Food Safety Authority (EFSA). Another objective of this study was to optimise the sample digestion process using microwave-assisted digestion with various reagents and reagent mixtures to ensure accurate and reliable measurements of elemental content.

## 2. Materials and Methods

### 2.1. Chemicals

The following chemicals were used during sample preparation and measurements:

- ultra-pure nitric acid, HNO_3_ conc. (*w* = 60%) (Merck, Darmstadt, Germany);
- hydrogen peroxide, H_2_O_2_ conc. (*w* = 30%) (Gram-mol, Zagreb, Croatia);
- standard multi-element solution VI of spectral purity for ICP (*γ* = 1000 mg/L) (Merck, Darmstadt, Germany);
- high-purity deionised water from inhouse facility, specific resistance ≥ 18 MΩ cm^−1^;
- Rh, internal standard solution (*γ* = 1 μg/L) (Merck; Darmstadt; Germany);
- NIST SRM 1547—peach leaves;
- NIST SRM 1573a—tomato leaves.

All laboratory glass- and plasticware was pre-cleaned with nitric acid (*w* = 2%).

### 2.2. Instruments

#### 2.2.1. Microwave Digestion System

Microwave-assisted digestion of all samples was performed using the ETHOS X Microwave Extraction System for Environmental Laboratories by Milestone Srl (Au, Switzerland). The pre-programmed method “Dried Plant Tissue program” was selected for this investigation. The total digestion time was 55 min, consisting of two steps: 15 min at 170 °C and 40 min at 200 °C.

#### 2.2.2. ICP-MS

Elemental analysis was performed using an inductively coupled plasma mass spectrometer (Agilent 7500cx ICP-MS; Agilent Tokyo, Japan). The working conditions for the validated method used are provided below in Table 1.

### 2.3. Sample Description and Pretreatment

A total of six samples were analysed. All mango samples were commercially available on the Croatian market. Four bags of dried mango from different producers were purchased (samples M1–M4). The countries of origin of the purchased samples were Burkina Faso and Ghana. One fresh mango originating from Brazil was also purchased. The fresh mango sample was separated into the mango flesh (SM) and the peel (P). All samples were cut into small pieces and dried in an oven at 60 °C for 24 h. The samples were weighed before and after drying, and the respective masses and the percentage of mass lost (moisture content) were calculated. All dried samples were ground in a mortar to homogenise them prior to microwave-assisted digestion. The list of samples and their characteristics are given in Table 2. No specific information on mango variety was given on the products.

### 2.4. Microwave-Assisted Digestion Procedure

Approximately 200 mg (to the nearest 0.1 mg) of each sample was weighed. The samples were transferred to Teflon PTFE vessels for microwave-assisted digestion. The vessels were pre-cleaned with nitric acid (*w* = 2%) before each digestion run. The digestion was carried out in four series with different volume ratios of nitric acid and hydrogen peroxide solution to optimise the digestion conditions using mango flesh samples. The mixtures used are shown in Table 3. These digestions were performed with sample M1 in duplicates.

Blank solutions were prepared in the same manner as the sample for each series and were digested in the same run in the microwave digestion system.

Series 3, which was shown to be the most appropriate digestion mixture, was then used to treat all samples (M1–M4, SM, P). Certified reference materials and blank solutions underwent the same procedure.

After digestion, all solutions were filtered using GE Healthcare Whatman Grade 42 Blue Ribbon filter paper, transferred to 25 mL volumetric flasks and filled up to the mark with ultra-pure water. The flasks were stored in a dry and dark place until analysis. Prior to analysis, all solutions were diluted 1:10 with ultrapure water, and the internal standard solution containing Rh was added with its final concentration of 10 µg/L.

### 2.5. Data Evaluation and Treatment

All analyses were performed in triplicate, and mean values were used for further evaluation and discussion.

Data were stored in Microsoft Office 365 Excel^®^. For statistical analysis and visualisation, R packages (R 4.3.2) with R-Studio were used. Hierarchical cluster analysis (HCA) and principal component analysis (PCA) were applied to explore dataset similarities and correlations. Euclidean distances and the Ward method were used for clustering, producing a dendrogram and heatmap. PCA highlighted systematic data variations.

Values below the limit of detection (LOD) were treated as zero. This approach was chosen since substituting by LOD/2 assumes that every non-detect is present at half the detection limit, which systematically inflates concentrations and can bias results upward. Treating non-detects as zero is more conservative to avoid overestimating true exposure or contamination. Zero treatment avoids introducing pseudo-values that do not come from actual measurements.

The Kruskal–Wallis H test was used for statistical differences, with post hoc analysis via pairwise Wilcoxon test. Adjustments were made using the Benjamini–Hochberg method, with significance at *p* < 0.05. Pearson correlation assessed variable relationships. These methods have been effective in similar studies [24,25].

## 3. Results and Discussion

### 3.1. Optimisation of the Digestion Procedure

Optimising the microwave-assisted digestion process is crucial before ICP-MS analysis. Proper optimisation ensures the sample is effectively prepared for instrumental analysis, leading to accurate and reliable results. The selection of appropriate reagents based on the sample’s nature and the elements of interest, along with optimising their concentrations and ratios, can significantly impact the digestion efficiency and process speed. The reagents used included concentrated nitric acid, a mixture of concentrated nitric acid and hydrogen peroxide, diluted nitric acid, and a mixture of diluted nitric acid and hydrogen peroxide. The digestion was performed in four series under the conditions listed in the Section 2. The elemental mass fractions in the sample M1 after applying different digestion mixtures are presented in Table 4.

**Figure 1:**
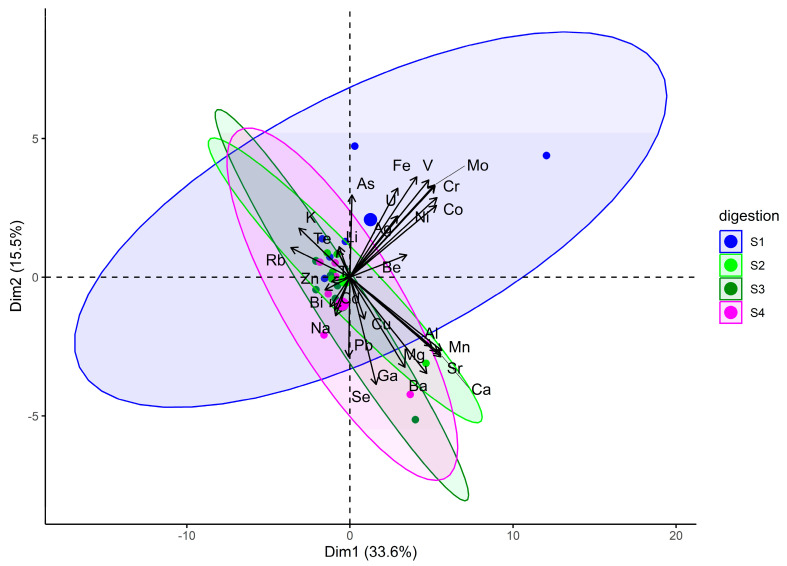
PCA biplot for digestion series (4) with ellipses around series for all samples (including flesh and peel, *n* = 24).

To determine whether statistically significant differences exist between the digestion series S1–S4, paired *t*-tests were performed for the following digestion combinations: S1 vs. S2, S2 vs. S3, S3 vs. S4, S1 vs. S4, S1 vs. S3, and S2 vs. S4. Using a significance level (α) of 0.01, only digestion series S1 differed significantly from the others, which on the other hand were not significantly different from each other (S2–S4). To further assess the optimisation of digestion, a chemometric method—Principal Component Analysis (PCA)—was used to identify significant differences in elemental mass fractions across different digestion methods. Since PCA optimises the distance between samples, results were interpreted to identify patterns or differences between digestion series. Visualisation involved plotting data points in a reduced-dimensional space defined by the chosen principal components, with each point representing a sample. This allowed for visualising similarities or differences between digestion series. The PCA results are shown in Figure 1, providing insights into the variability of elemental mass fractions and potential patterns or differences between digestion series.

The first two dimensions of the PCA in Figure 1 represent 49% of the variance. The vectors represent the principal components (chemical elements) obtained from the decomposition of the covariance matrix into eigenvalues. Each vector indicates the direction of the greatest variance in the data. The first vector (PC1) shows the direction of the highest variance (here 33.6%); the second vector (PC2) indicates the direction of the second highest variance (here 15.5%); followed by PC3 (here 12.6%), PC4 (here 8.5%), PC5 (here 6.6%), PC6 (5.6%), and PC 7 to PC10 with less than 5% each. Variables (elemental mass fractions) that strongly contribute to a principal component will have larger points and align more closely with the direction of the corresponding vector. Based on the results shown in Figure 1, it can be concluded that digestion S1 differed significantly from the other series (S2–S4), which did not significantly differ from each other.

Ellipses around the origin represent the “contribution” or “importance” of each variable (elemental mass fraction) to the principal components (chemical elements). A larger circle indicates a more influential variable in determining the direction and magnitude of the principal component vector. Variables near the edge of the circle significantly contributed to the corresponding principal component, while those closer to the centre contributed less. Points far from the main cluster may represent samples with unique elemental compositions, as shown for the S1 digests in Figure 1. The distance between points reflects similarity or difference between samples. Points close to each other had similar compositions, while distant points were different. The position of each point along the principal component vectors represents the projection of the original data onto the reduced-dimensional space defined by the principal components.

In series S1, three elements stand out. Two blue points were near the edges of the blue circle, indicating that these samples significantly contributed to the corresponding principal components, arsenic and nickel, suggesting they had different elemental compositions compared to other digests. The mass fractions of nickel and arsenic in these S1 digests were much higher than in other S1 samples. A larger blue point aligned more closely with the direction of the uranium vector and significantly contributed to the principal component; it was observed that one digest solution of S1 had a much higher uranium mass fraction compared to the others. Other blue points in series S1 were close together, indicating similar compositions. The same applied to the other series S2, S3, and S4. In S2, a digest with higher Mg content deviated significantly. In S3, a digest with a significantly higher Ga mass fraction is noted. In S4, some digests had higher Na and Ga content than others.

The PCA diagram indicates a significant difference between digestion S1 and digestions S2, S3, and S4, which did not differ significantly from each other. Therefore, series S1 (concentrated nitric acid) was not considered when selecting the optimal reagent combination for digesting dried mango samples. Given that the best sample quality and quick attainment of a clear solution with minimal residue was achieved with series 3 (6 mL concentrated nitric + 3 mL H_2_O_2_ (30%)), this digestion mixture was further used to treat all other samples as well as certified reference materials prior to elemental analysis by ICP-MS.

### 3.2. Applicability of the Analytical Method

Prior to the analysis of all samples, the accuracy (precision and trueness) of the ICP-MS method was determined using two certified reference materials, namely, NIST SRM 1547—*Peach leaves* and NIST SRM 1573a—*Tomato leaves*. Detection limits (LOD) ranged from 0.001 mg/kg to 0.2 mg/kg, except for Na, K, and Ca with values up to 2.6 mg/kg (see Table S1). The correlation coefficient for all calibration curves was at least 0.998, and precision, expressed as RSD, was less than 4% for all analytes. The results obtained for the two reference materials after performing digestion using mixture S3 are listed in Table 5. Except for Se, all recoveries were in the acceptable range. Thus, the applicability of the used method was proven.

### 3.3. Elemental Composition of Mango

Elemental analysis was conducted on commercially available dried mango samples M1–M4, as well as dried fresh mango (SM) and its peel (P). A total of 29 elements were determined. The mean values calculated for all samples are given in Table 6. Table S2 in the Supplementary Information contains additionally the RSD for all data alongside literature values for comparison as a summarised overview, whereas a detailed discussion on the analytes follows here.

Macroelements, like K, Mg, Ca, and Na, play an important role in the human body, and thus daily diet should contain sufficient amounts of these elements. Mango flesh as well as peel are rich in these elements. The most abundant element in the commercially available dried mango samples (M1-M4) was potassium, ranging from 6600 mg/kg to 10,699 mg/kg. Magnesium was the next most prevalent, ranging from 418 mg/kg to 639 mg/kg, followed by calcium, which ranged from 251 mg/kg to 360 mg/kg, and sodium, which ranged from 48.1 mg/kg to 551 mg/kg. A significant difference was observed in the sodium content of sample M4 compared to the other samples. Some manufacturers add salt to dried mango and other dried fruits as a flavour enhancer or preservative. Salt not only enhances flavour but also acts as a preservative to extend the product’s shelf life [26]. The use of salt or sodium-containing solutions in the processing and preservation stages can increase the sodium content, which could explain its very high concentration in sample M4. Apart from the findings for M4, the industrial drying process did not seem to have a significant impact on the contents of macro and trace elements. The data obtained for the fresh fruit were in a similar order of magnitude, only the Ca content was higher in SM than in M1-M4. Considering a single serving of commercially available dried mango flesh of 40 g and the recommend daily intake for the macroelements [27], 10% to 16% of potassium, 4.0% to 8.0% of magnesium, 0.20% to 1.5% of sodium, and 1.0% to 1.5% of calcium can be covered (for more details, see Table S3). Monro and coworkers found lower amounts of all macroelements in mango from Tonga, namely, 13 mg/kg for Na, 1080 mg/kg for K, 160 mg/kg for Ca, and 100 mg/kg for Mg [28]. Macroelemental contents can also be influenced by different post-harvest treatments, as shown by Khan et al. [29].

**Figure 2:**
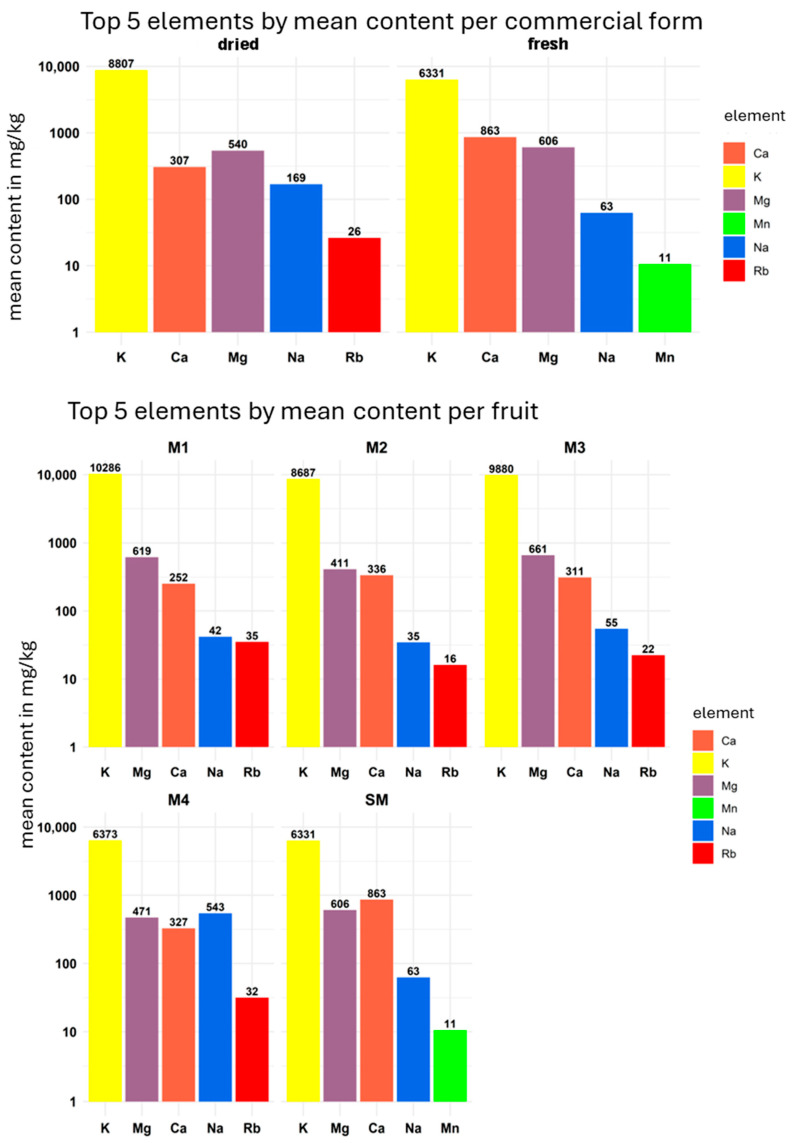
Top five element content of mango flesh of all replicates for the samples M1, M2, M3, M4, and SM, as well as for dried and fresh in comparison.

**Figure 3:**
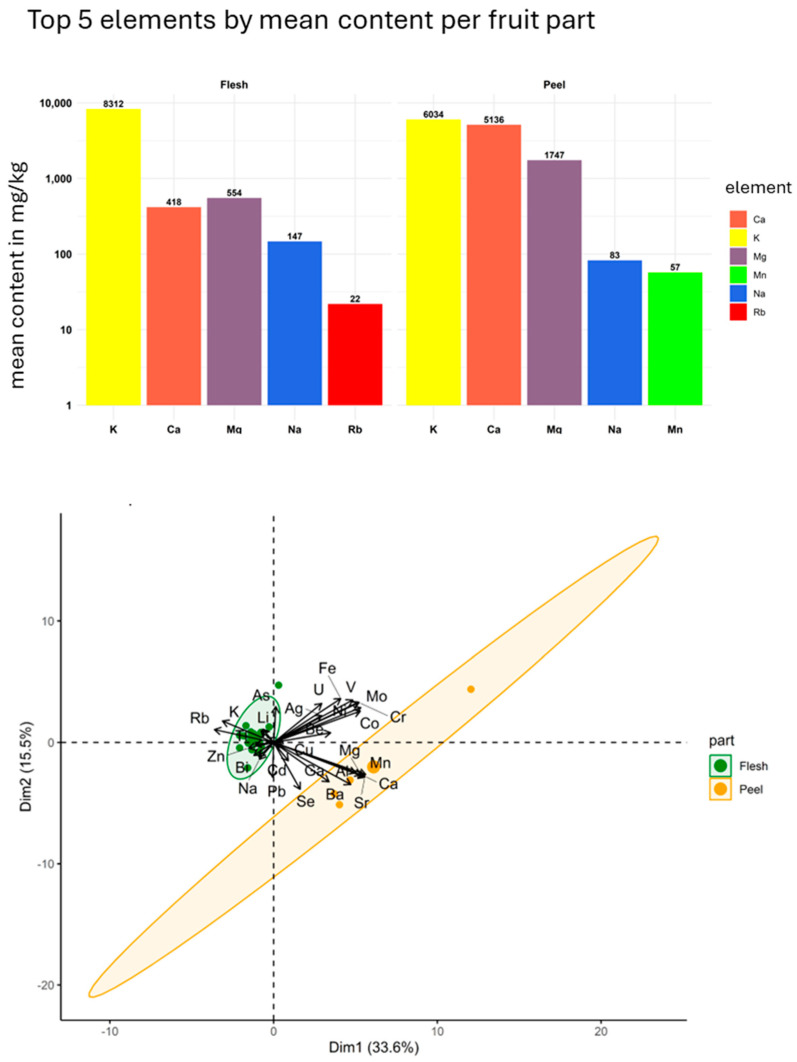
(**Above**): Top five element content for flesh and peel. (**Below**): PCA biplot of all samples (*n* = 24) with ellipses around the two parts, flesh and peel.

In Figure 2 and Figure 3, the contents of the top five elements—expressed as mean values—in the analysed mango samples are presented separated by commercial form (dried or fresh), fruit (M1–M4 and SM), and fruit part (flesh and peel), allowing for easier visual comparison of the different sample groups.

Trace elements or micronutrients are elements present in very small amounts. When discussing the results of mango analysis, these elements were divided into three groups: micronutrients (Cr, Mn, Fe, Cu, Zn, Se, and Mo), those that currently have no known physiological role (V, Li, Co, Ba, Rb, Sr, Bi, and Te), and those that are potentially toxic to the human body (Al, Tl, Be, Ag, Ga, As, Cd, Ni, Pb, and U). Mango plants require Mn, Zn, B, Cu, Mg, and Mn for healthy growth, with zinc being the most essential for plant growth and leaf size [30].

Regarding micronutrients, chromium, iron, and molybdenum were below the detection limits (<LOD) in all samples. Selenium was also below detection limits for samples M1 and M2, while its mass fraction in samples M3 and M4 was equal (0.020 mg/kg) and very low, close to the LOD. This finding is in contrast to the literature, where four types of mango in Yanbian County of Panzhihua are reported to be naturally high in selenium, providing a basis for scientific selenium reinforcement [31]. The low iron content is of importance for methane production based on mango. Adding trace elements, Ni and Fe, to mango-peel-fed digesters improves biogas yield and methane content, with iron-fed digesters yielding the highest methane content [32].

Manganese was generally the most abundant essential micronutrient. The highest content of Mn was in sample M2 (11.0 mg/kg), followed by M4 (9.33 mg/kg) and M1 (8.01 mg/kg), while the content in sample M3 was half that of the other samples (4.44 mg/kg). Zinc was the second most abundant essential micronutrient. Sample M2 stood out with a very low zinc content (1.85 mg/kg). This sample had almost four times less Zn than samples M1 (6.92 mg/kg) and M4 (7.15 mg/kg). Sample M3 had the highest zinc content (9.51 mg/kg), which was five times higher than in sample M2. Regarding the micronutrient copper, all samples had relatively similar contents, with M1 having the highest one (4.73 mg/kg), followed by M3 (4.19 mg/kg), M4 (3.76 mg/kg), and M2 (2.52 mg/kg). Obediah and colleague found the trace mineral elements Co, Fe, Mn, Ni, and Zn in low mass fractions (0.006 mg/kg up to 0.1 mg/kg) in mango and other five tropical fruits commonly consumed in Port Harcourt, Nigeria [33].

As for the macroelements, the contribution of a 40 g serving unit of dried mango to cover the needs of Cu, Mn, and Zn was calculated; the percentages of the daily required intake covered ranged from 11 to 42%, 8 to 25%, and 0.7 to 4.7%, respectively (for more details, see Table S3).

Dried fresh mango and its peel had relatively similar levels of essential micronutrients as commercially available dried mango samples. Chromium, iron, and molybdenum were below detection limits in both the SM and P samples, as well as in samples M1–M4. The copper content was very similar across all samples, with 4.16 mg/kg in the SM sample and 3.88 mg/kg in the P sample. Zinc was less prevalent compared to the commercially available samples (except for the M2 sample), with levels of 2.84 mg/kg in fresh mango and 3.65 mg/kg in the peel. In the SM sample, selenium was below detection limits, as in the M1 and M2 samples, while the P sample contained the highest amount of Se among all the samples, at 0.256 mg/kg, which was almost 13 times higher than in the M3 and M4 samples. Chromium and selenium are trace minerals that were generally found in smaller quantities in many foods, and their presence in mango may be limited. Manganese was the most abundant element in these two samples. In the SM sample, the Mn content did not differ much from samples M1-M4, but it was slightly higher (10.3 mg/kg), while a very high amount of Mn was found in the peel, as much as 57.7 mg/kg, which was about 13 times more than in, for example, the M3 sample. Monro et al. not only analysed the content of macroelements in fresh mango but also determined the levels of the following essential micronutrients in fresh mango: Cu: 1.9 mg/kg, Fe: 27.8 mg/kg, Mn: 1.4 mg/kg, and Zn: 1.3 mg/kg [28]. The differences were supposed to be caused by the different origin and harvesting time, but also it needs be considered that the analysis by Monro was performed in the 1980s. As for macronutrients, the consumption dried mango leads to a higher supply of micronutrients, such as Cu, Mn, and Zn, compared to consuming fresh mango.

Dried mango samples also contained trace elements that are non-essential but are not toxic unless present in excessive amounts. This group includes Li, V, Co, Ga, Rb, Sr, Te, and Bi. Cobalt, tellurium, and vanadium were below the detection limits in all samples. The most prevalent element in all commercially available dried mango samples was Rb. The highest content was found in sample M1 (36.6 mg/kg), while sample M2 contained nearly half of that (16.3 mg/kg); in between were sample M3 (23.3 mg/kg Rb) and sample M4 (33.0 mg/kg Rb). Rubidium is a natural element that occurs in very low concentrations in the Earth’s crust. It can be present in soil and water, and plants can absorb trace amounts of rubidium from the soil as they grow. The concentration of rubidium in food depends on factors such as the rubidium content of the soil in which the plants are grown and the specific type of food. Rubidium does not have a known biological function in the human body, and in this respect, it is classified as strontium, gallium, lithium, and tellurium. The Sr content was almost the same in samples M2 and M4 (2.25 mg/kg and 2.39 mg/kg), followed by sample M3 (1.25 mg/kg) and sample M1 (0.828 mg/kg). There were no significant differences in the gallium content between the samples, covering a range from 0.027 mg/kg to 0.121 mg/kg. Regarding Bi, M3 contained the most (1.22 mg/kg), followed by M1 (0.454 mg/kg), M2 (0.062 mg/kg), and M4 (<LOD). Lithium was below the detection limit in samples M2 and M4 but was found in traces in samples M1 and M3 (0.013 mg/kg and 0.050 mg/kg). These trace elements were present in very small amounts, and their intake is not considered essential for the human body. Since such elements are generally not the focus of nutritional analysis or dietary recommendations, there is no data available or scientific literature for comparison. It is thus supposed that these elements will not exhibit any harmful effect on the body when consuming the (dried) mango. Having a closer look on samples SM and P, the elements V, Co, and Li were below detection limits. In sample SM, Ga had a very similar mass fraction (0.047 mg/kg) as in samples M1–M4, while in sample P, it was about four times higher (0.196 mg/kg). Rb was by far the most abundant element in samples M1–M4, while in samples SM and P, it was about eight times lower (4.41 mg/kg and 4.20 mg/kg, respectively) when compared to sample M1. On the other hand, the content of Sr was much higher: compared to sample M1, Sr was nearly six times higher in sample SM (4.70 mg/kg), and as much as 27 times higher in sample P (22.6 mg/kg). The content of bismuth was comparable in sample SM (0.576 mg/kg) and sample P (0.571 mg/kg).

The final group of elements analysed consisted of potentially toxic and toxic trace elements. Some elements are toxic in their elemental state, while others form compounds that are poisonous. The elements that are toxic in their elemental form or in compounds.

The most prevalent potentially toxic element in the commercially available dried mango samples (M1–M4) was aluminium. The highest mass fraction of Al was found in sample M2 (8.28 mg/kg), followed closely by M3 (7.49 mg/kg), then M4 (6.19 mg/kg), and finally M1 with the lowest Al content (4.96 mg/kg). Cadmium was present in samples M1, M3, and M4, while it was below the detection limit in sample M2. The mass fraction of cadmium in sample M3 was 0.042 mg/kg, which was 22 times higher than in sample M1 (0.0008 mg/kg) and 21 times higher than in sample M4 (0.0017 mg/kg). Lead was below the detection limit in all samples except M3, where the Pb content was 0.058 mg/kg. A positive finding is that arsenic, nickel, and thallium were below the detection limit in all samples.

Barium had by far the highest mass fraction. It was present in all samples, ranging from 1.08 to 5.17 mg/kg. The mass fraction of beryllium was very small—0.001 mg/kg in sample M1, and six times higher in sample M4 (0.006 mg/kg), while it was below the detection limit in samples M2 and M3. Silver was quantified only in sample M2, with a mass fraction of 0.077 mg/kg. Small and nearly equal amounts of uranium were found in samples M1 and M3 (0.001 mg/kg and 0.002 mg/kg, respectively), while in samples M2 and M4, it was below the detection limit.

The literature review mentioned the permitted levels for certain contaminants in fruit, as established by EFSA (lead—0.1 mg/kg, cadmium—0.05 mg/kg, arsenic—0.2 mg/kg) [34]. Arsenic was below the detection limit in all samples. Cd was within the acceptable range in samples M1, M2, and M4, while in sample M3 (0.042 mg/kg), it was close to the permissible limit. It is possible that the sample was contaminated during processing, which could explain the slightly higher Cd content. Sample M3 was the only one that contained lead, but the amount was below the given limit (0.058 mg/kg < 0.1 mg/kg). Uranium can enter food through various pathways. It may be naturally present in soil as a result of geological processes or introduced by human activities such as mining or the use of certain fertilisers. Plants can absorb uranium from soil as they grow [35,36]. Water sources like rivers and lakes may contain trace amounts of uranium, which plants can absorb through their roots.

Similar reasons may also explain the presence of aluminium and barium [37,38]. Al is naturally present in the Earth’s crust, and its content in soil can vary depending on the geological characteristics of the region where the mango is grown. The use of certain aluminium-containing fertilisers or soil amendments in agriculture, as well as industrial activities, may contribute to Al content.

There was no significant difference in the mass fractions of the potentially toxic elements analysed between samples M1–M4 and samples SM and P. Be, As, Ag, Ni, and Tl were below detection limits. Al was the most abundant potentially toxic element in all samples, and its level in the dried mango peel was significantly higher than in the other samples. The Al content in sample P was 61.8 mg/kg, which was as much as 10 times higher than in sample M4, and much higher than in sample SM, given that the Al mass fractions in samples M4 and SM were nearly identical (6.19 mg/kg and 6.28 mg/kg, respectively). Cd was present in small amounts in both samples, and its mass fraction did not differ significantly from the other samples (0.0036 mg/kg and 0.0059 mg/kg). The Ba content in sample SM did not differ significantly from samples M1–M4 (2.02 mg/kg); this was about twice as much as in sample M1, but about half as much as in sample M4. The peel of dried mango was found to be rich in barium, with a slightly higher content than in samples M1–M4 (7.55 mg/kg). In all commercially available dried mango samples, except for sample M3, Pb was below detection limits. In samples P and M3, Pb content was nearly identical (0.056 mg/kg and 0.058 mg/kg, respectively). In sample SM, lead was present much less (0.0036 mg/kg). The uranium mass fraction in sample SM was identical to that in sample M1 (0.001 mg/kg), while in sample P, it matched that in sample M3, at 0.002 mg/kg.

Generally, sources of toxic element accumulation in mango fruits may arise from excessive use of fertilisers and pesticides during the early stages of cultivation, as well as from environmental contamination in industrial regions where emissions, wastewater, and soil deposition contribute to elevated metal levels [39]. Furthermore, the quality of irrigation water influences metal levels in soil and fruits, as shown by Anjum and coworkers for the application of sewage water on mango trees [40], which led to elevated Cu and Ni contents in the leaves and fruits.

Since no variety was stated for the analysed samples, this aspect in elemental uptake and accumulation cannot be assessed. However, a study by Siric and colleague on *Mangifera indica* L. var. Dasheri and Langra did not find significant differences in the contents of four toxic elements (Cd, Cr, Pb, and As) [39]. This contrasts with the findings by Bi et al., who found significantly different contents for Cd and Pb in six mango cultivars [41].

## 4. Conclusions

Detailed elemental analysis revealed that in both commercially available dried mango samples and dried fresh mango, all potentially toxic elements were present in content well below the permitted limits, indicating that the samples are safe for consumption. There were significant differences in the elemental content between the flesh and the peel for certain elements. Differences were evident for both essential and toxic elements. By removing the peel, it is possible to reduce the intake of, for example, Cd and Pb, but also of Ca, Mg, Zn, and Se. The elemental analysis confirmed that dried mango is a good source of essential elements, particularly Ca, K, Mg, and Mn. Toxic elements such as Cd, Pb, and As, which could pose a risk to human health, were not found in concentrations above the permitted limits. A small portion of dried mango can significantly contribute to the recommended daily allowance (RDA) of essential elements, and its high nutritional value makes dried mango an excellent and healthy snack.

For further analysis of mango samples, it is necessary to conduct research on a higher number of both fresh and dried mango samples, as well as on individual parts such as the mesocarp, peel, and seed. Another interesting point would be the analysis of different mango varieties and to investigate their elemental composition.

## Figures and Tables

**Table 1:** Optimised ICP-MS operating conditions.

Parameter	Condition
RF power	1500 W
Carrier gas flow (Ar)	0.90 L/min
Additional gas flow (Ar)	0.20 L/min
Nebulizer	MicroMist
Nebulizer pump	0.10 rps
Spray chamber	Scott double pass
Integration time	0.10 s
Repeated samples	Two
Calibration	External
Calibration solution	Multi-element VI (Merck)
Isotopes	^7^Li, ^9^Be, ^23^Na, ^24^Mg, ^27^Al, ^39^K, ^43^Ca, ^51^V, ^53^Cr, ^55^Mn, ^56^Fe, ^59^Co, ^60^Ni, ^63^Cu, ^66^Zn, ^69^Ga, ^75^As, ^82^Se, ^85^Rb, ^88^Sr, ^95^Mo, ^107^Ag, ^111^Cd, ^125^Te, ^137^Ba, ^205^Tl, ^208^Pb, ^209^Bi, ^238^U
Internal standard (10 µg/L)	^103^Rh
Collision chamber	Usage depending on analyte

**Table 2:** Sample characterisation.

Sample	Designation	Country	Water Content in %
Dried Mango 1	M1	Burkina Faso	17.31%
Dried Mango 2	M2	Burkina Faso	15.15%
Dried Mango 3	M3	Burkina Faso	14.75%
Dried Mango 4	M4	Ghana	15.00%
Fresh Mango	SM	Brazil	85.10%
Fresh Mango Peel	P	Brazil	77.49%

**Table 3:** Reagent mixtures for digestion optimisation.

Digestion Series Name	Designation	Digestion Mixture
Series 1	S1	6 mL conc. nitric acid
Series 2	S2	6 mL diluted nitric acid (7 mol/L)
Series 3	S3	6 mL conc. nitric acid + 3 mL H_2_O_2_ (30%)
Series 4	S4	6 mL diluted nitric acid (7 mol/L) + 3 mL H_2_O_2_ (30%)

**Table 4:** Mass fractions of all quantified analytes in sample M1 after digestion using different reagent mixtures (digestion series S1–S4) in mg/kg.

Element	S1	S2	S3	S4
Li	0.053	0.006	0.013	<LOD
Be	<LOD	<LOD	0.001	0.001
Na	53.4	37.0	76.8	<LOD
Mg	588	628	639	620
Al	<LOD	2.85	4.96	<LOD
K	9711	10,387	10,699	10,347
Ca	227	310	251	222
V	<LOD	<LOD	<LOD	<LOD
Cr	<LOD	<LOD	<LOD	<LOD
Mn	9.13	9.60	8.01	7.98
Fe	3.08	2.29	<LOD	<LOD
Co	<LOD	<LOD	<LOD	<LOD
Ni	<LOD	0.113	<LOD	<LOD
Cu	3.74	4.91	4.73	4.94
Zn	5.72	10.9	6.92	2.68
Ga	0.019	0.025	0.027	0.045
As	<LOD	<LOD	<LOD	<LOD
Se	<LOD	<LOD	<LOD	0.081
Rb	33.0	35.5	36.6	35.1
Sr	0.718	0.804	0.828	0.764
Mo	<LOD	<LOD	<LOD	<LOD
Ag	2.03	0.257	<LOD	<LOD
Cd	<LOD	<LOD	0.0007	<LOD
Te	<LOD	<LOD	<LOD	<LOD
Ba	0.741	1.085	1.082	0.740
Tl	<LOD	<LOD	<LOD	0.0003
Pb	<LOD	<LOD	<LOD	<LOD
Bi	<LOD	<LOD	0.454	1.44
U	<LOD	<LOD	0.0006	0.003

**Table 5:** Mass fractions for all analytes quantified in the digest solutions of the reference materials given in mg/kg alongside recovery.

Element	Results	Certified Value SRM 1547	Recovery in %	Results	Certified Value SRM 1573a	Recovery in %
Li	0.084	-	/	0.366	-	/
Be	0.016	-	/	0.017	-	/
Na	25.3	23.8 ± 1.6	106%	150.4	136.1 ± 3.7	110%
Mg	4168	4320 ± 150	96%	11,408	12,000	95%
Al	258.2	248.9 ± 6.5	104%	641	598.4 ± 7.1	107%
K	23,241	24,330 ± 380	95%	23,100	26,760 ± 480	86%
Ca	16,100	15,590 ± 160	103%	51,014	50,450 ± 550	101%
V	0.298	0.367 ± 0.038	81%	0.704	0.835 ± 0.034	84%
Cr	1.1	1	110%	2.12	1.988 ± 0.034	107%
Mn	95.3	97.8 ± 1.8	97%	240	246.3 ± 7.1	97%
Fe	214	219.8 ± 6.8	97%	378	367.5 ± 4.3	103%
Co	0.1	0.07	143%	0.593	0.5773 ± 0.0071	103%
Ni	0.867	0.689 ± 0.095	126%	1.20	1.582 ± 0.041	76%
Cu	3.54	3.75 ± 0.37	94%	4.62	4.70 ± 0.14	98%
Zn	16.9	17.97 ± 0.53	94%	31.8	30.94 ± 0.55	103%
Ga	3.32	-	/	4.40	-	/
As	0.073	0.062 ± 0.014	118%	0.129	0.1126 ± 0.0024	114%
Se	0.201	0.120 ± 0.017	167%	0.084	0.0543 ± 0.0020	155%
Rb	18.7	19.65 ± 0.089	95%	15.4	14.83 ± 0.31	104%
Sr	48.4	53.0 ± 5.0	91%	93	85	110%
Mo	0.0681	0.0603 ±0.0068	113%	0.42	0.46	91%
Ag	1.16	-	/	0.020	0.017	118%
Cd	0.0250	0.0261 ± 0.0022	96%	1.49	1.517 ± 0.027	98%
Te	1.5 × 10^−3^	-	/	0.001	-	/
Ba	111	123.7 ± 5.5	90%	26.8	-	/
Tl	0.061	-	/	0.063	-	/
Pb	1.01	0.869 ± 0.018	116%	1.12	-	/
Bi	0.067	-	/	0.012	-	/
U		-		0.018	0.035	51%

**Table 6:** Mean elemental content of mango flesh (M1-M4, SM) and peel (P) in mg/kg.

Element	Sample					
	M1	M2	M3	M4	SM	P
Na	76.7	48.1	81.8	551	70.2	40.9
K	10,699	8781	10,484	6600	6722	6194
Ca	251	354	328	360	921	5245
Mg	639	418	682	486	646	1771
Cr	<LOD	<LOD	<LOD	<LOD	<LOD	<LOD
Mn	8.01	11.0	4.44	9.33	10.3	57.7
Fe	<LOD	<LOD	<LOD	<LOD	<LOD	<LOD
Cu	4.73	2.52	4.19	3.76	4.16	3.88
Zn	6.92	1.85	9.51	7.15	2.84	3.65
Se	<LOD	<LOD	0.020	0.020	<LOD	0.256
Mo	<LOD	<LOD	<LOD	<LOD	<LOD	<LOD
Ni	<LOD	<LOD	<LOD	<LOD	<LOD	<LOD
V	<LOD	<LOD	<LOD	<LOD	<LOD	<LOD
Li	0.013	<LOD	0.050	<LOD	<LOD	<LOD
Co	<LOD	<LOD	<LOD	<LOD	<LOD	<LOD
Ga	0.027	0.083	0.045	0.121	0.047	0.196
Rb	36.6	16.3	23.3	33.0	4.41	4.20
Sr	0.828	2.25	1.25	2.39	4.70	22.6
Bi	0.454	0.062	1.22	<LOD	0.576	0.571
Te	<LOD	<LOD	<LOD	<LOD	<LOD	<LOD
Al	4.96	8.28	7.49	6.19	6.28	61.8
Tl	<LOD	<LOD	<LOD	<LOD	<LOD	<LOD
Be	0.001	<LOD	<LOD	0.006	<LOD	<LOD
Ag	<LOD	0.077	<LOD	<LOD	<LOD	<LOD
Ba	1.08	3.31	1.64	5.17	2.02	7.55
As	<LOD	<LOD	<LOD	<LOD	<LOD	<LOD
Cd	0.0008	<LOD	0.042	0.0017	0.0036	0.0059
Pb	<LOD	<LOD	0.058	<LOD	0.0039	0.056
U	0.001	<LOD	0.002	<LOD	0.001	0.002

## Data Availability

The original contributions presented in this study are included in the article/Supplementary Material. Further inquiries can be directed to the corresponding author.

## Supplementary Materials

The following supporting information can be downloaded at https://www.mdpi.com/article/10.3390/foods14173096/s1. Table S1: Validation parameter for the analytes in order of increased m/z (R, coefficients a and b of linear trendlines y = ax + b, with x being the molar concentration in the digest solution alongside standard deviation for a and b; recovery as mean of different CRMs where applicable; RSD per sample; LOD as mass fraction in the original sample). Table S2: Mean elemental content of mango flesh and peel in mg/kg alongside literature data. RSD in % is given in parentheses for the determined values. Table S3: Comparison of the mass of elements in 40 g of dried mango with the RDA values.
