# Supplementary material for: Elemental Analysis and Chemometric Assessment of Edible Part and Peel of Mango Fruits (Mangifera indica L.)
Source: Foods. 2025 Sep 3;14(17):3096. doi: 10.3390/foods14173096 (PMC12428084; doi:10.3390/foods14173096)
Supplement: Supplementary file 1 [file foods-14-03096-s001.zip › foods-3814304-supplementary.pdf]

Table S1: Validation parameter for the analytes in alphabetical order (R, coefficients a and b of linear trendlines y = a x + b, with x being the molar concentration in the digest solution; recovery as mean of different CRMs where applicable; RSD per sample).

Analyte	R	a	b	Recovery in %	RSD in %	LOD ω in mg/kg
Li	0.999983558	0.163059122	0.001006889	Not certified	3.6	0.0049
Be	0.999989762	0.045952017	7.19456E-05	Not certified	2.2	0.00092
Na	0.999976289	0.288441549	-0.199732787	108	1.3	2.2
Mg	0.999979123	0.204618216	0.000153732	96	1.0	0.032
Al	0.999989798	0.256593103	-0.000493816	105	1.0	0.068
K	0.999985354	0.003427897	0.00438771	90	1.1	2.6
Ca	0.999999935	0.000639921	0.000216485	102	1.8	0.91
V	0.999996027	0.085774728	0.000127107	83	1.6	0.0079
Cr	0.999986922	0.015549035	0.000127763	108	3.2	0.15
Mn	0.999998166	0.633056343	-0.00093609	97	1.0	0.0025
Fe	0.999999324	0.111528699	0.00222414	100	0.8	0.069
Co	0.999998145	0.511266922	-0.000756289	123	2.7	0.0027
Ni	0.999999797	0.106857636	-0.000286773	101	2.3	0.0035
Cu	0.99999992	0.19241668	1.01669E-06	96	1.1	0.028
Zn	0.999999558	0.090344487	0.000249423	98	1.1	0.015
Ga	0.999996857	0.460805233	0.000536972	Not certified	2.4	0.0025
As	0.999998134	0.015032284	0.000190875	116	2.6	0.068
Se	0.999991579	0.0006143	-0.000190721	161	2.5	0.0014
Rb	0.999999865	0.445758359	0.000681775	99	1.5	0.00079
Sr	0.999998243	0.718056815	0.000431282	100	1.2	0.00030
Mo	0.999995281	0.131265332	-0.000358533	97	1.8	0.0028
Ag	0.999998859	0.4423623	0.000106276	Not certified	2.9	0.0022
Cd	0.99999866	0.1008354	-0.000107963	97	1.5	0.00047
Te	0.999997243	0.014239257	-7.19751E-05	Not certified	2.4	0.00022
Ba	0.999999979	0.12312127	-8.82024E-07	90	1.5	0.0046
Tl	0.999999229	1.28326397	-0.000249022	Not certified	1.9	0.0012
Pb	0.999999567	0.438024509	9.16252E-05	116	1.7	0.0026
Bi	0.999687731	1.069512424	-0.011314813	Not certified	3.9	0.00061
U	0.999996793	2.071021723	-0.000178919	51	3.1	0.00018

Table S2: Mean elemental content of mango flesh and peel in mg/kg alongside literature data. RSD in % is given in parentheses for the determined values.

Element	Sample						Monro et al.	Obediah and Elechi-Amadi	Siric et al.	Anjum et al.
	M1	M2	M3	M4	SM	P	[28]	[33]	[39]	[40]
Na	76.7 (2.1)	48.1 (1.9)	81.8 (1.2)	551 (0.58)	70.2 (0.76)	40.9 (2.1)	13			
K	10699 (0.76)	8781 (0.73)	10484 (0.33)	6600 (0.71)	6722 (0.95)	6194 (0.82)	1080			
Ca	251 (2.5)	354 (5.6)	328 (1.3)	360 (1.2)	921 (0.93)	5245 (1.8)	160			
Mg	639 (1.9)	418 (2.3)	682 (1.6)	486 (1.2)	646 (0.85)	1771 (2.4)	100			
Cr	<LOD	<LOD	<LOD	<LOD	<LOD	<LOD			0.11 – 0.80	
Mn	8.01 (1.0)	11.0 (2.3)	4.44 (0.92)	9.33 (0.39)	10.3 (0.61)	57.7 (1.8)	1.4	0.0051		
Fe	<LOD	<LOD	<LOD	<LOD	<LOD	<LOD	27.8	0.101		
Cu	4.73 (0.80)	2.52 (1.1)	4.19 (1.8)	3.76 (1.7)	4.16 (0.36)	3.88 (1.6)	1.9			1.73 – 6.74
Zn	6.92 (1.4)	1.85 (2.6)	9.51 (1.5)	7.15 (1.4)	2.84 (1.1)	3.65 (1.7)	1.3	0.0384		< LOD
Se	<LOD	<LOD	0.020 (14)	0.020 (10)	<LOD	0.256 (4.6)				
Mo	<LOD	<LOD	<LOD	<LOD	<LOD	<LOD				
Ni	<LOD	<LOD	<LOD	<LOD	<LOD	<LOD		0.006		1.25 – 21.16
V	<LOD	<LOD	<LOD	<LOD	<LOD	<LOD				
Li	0.013 (5.4)	<LOD	0.050 (2.9)	<LOD	<LOD	<LOD				
Co	<LOD	<LOD	<LOD	<LOD	<LOD	<LOD		0.0156		
Ga	0.027 (8.7)	0.083 (4.6)	0.045 (10)	0.121 (7.2)	0.047 (2.6)	0.196 (4.9)				
Rb	36.6 (0.99)	16.3 (3.3)	23.3 (1.2)	33.0 (1.6)	4.41 (0.89)	4.20 (1.4)				
Sr	0.828 (2.9)	2.25 (2.3)	1.25 (2.1)	2.39 (2.7)	4.70 (1.6)	22.6 (1.7)				
Bi	0.454 (4.3)	0.062 (3.1)	1.22 (3.8)	<LOD	0.576 (9.6)	0.571 (1.8)				
Te	<LOD	<LOD	<LOD	<LOD	<LOD	<LOD				
Al	4.96 (1.1)	8.28 (2.1)	7.49 (1.0)	6.19 (1.1)	6.28 (0.94)	61.8 (1.3)				
Tl	<LOD	<LOD	<LOD	<LOD	<LOD	<LOD				
Be	0.001 (33)	<LOD	<LOD	0.006 (6.6)	<LOD	<LOD				
Ag	<LOD	0.077 (1.4)	<LOD	<LOD	<LOD	<LOD				
Ba	1.08 (3.1)	3.31 (3.4)	1.64 (3.6)	5.17 (2.7)	2.02 (2.3)	7.55 (1.4)				

Element	Sample						Monro et al. [28]	Obediah and Elechi-Amadi [33]	Siric et al. [39]	Anjum et al. [40]
	M1	M2	M3	M4	SM	P				
As	<LOD	<LOD	<LOD	<LOD	<LOD	<LOD			0.01 – 0.10	
Cd	0.0008 (49)	<LOD	0.042 (23)	0.0017 (29)	0.0036 (15)	0.0059 (8.6)			0.02 – 0.08	< LOD
Pb	<LOD	<LOD	0.058 (5.2)	<LOD	0.0039 (3.5)	0.056 (2.4)			0.02 – 0.15	
U	0.001 (22)	<LOD	0.002 (19)	<LOD	0.001 (11)	0.002 (8.4)				

Table S3: Comparison of the mass of elements in 40 g of dried mango with the RDA values.

Element	RDA in mg/day [27]		Percentage per 40 g Serving of Dried Mango in %	
	**Men**	**Women**	**Men**	**Women**
Na	1500	1500	0.20 – 1.5	0.20 – 1.5
K	3400	2600	7.8 – 12.6	10.2 – 16.5
Ca	1000	1000	1.0 – 1.4	1.0 – 1.4
Mg	420	320	4.0 – 6.5	5.2 – 8.5
Cr	0.035	0.025	-	-
Mn	2.3	1.8	7.7 – 19.1	10.0 – 24.5
Fe	18	8	-	-
Cu	0.9	0.9	11.2 – 41.5	11.2 – 41.5
Zn	11	8	0.7 – 3.5	0.9 – 4.7
Se	0.055	0.055	≤ 1.5	≤ 1.5
Mo	0.045	0.045	-	-
